# Cruzipain and Its Physiological Inhibitor, Chagasin, as a DNA-Based Therapeutic Vaccine Against *Trypanosoma cruzi*

**DOI:** 10.3389/fimmu.2020.565142

**Published:** 2020-10-09

**Authors:** Natacha Cerny, Augusto Ernesto Bivona, Andrés Sanchez Alberti, Sebastián Nicolás Trinitario, Celina Morales, Alejandro Cardoso Landaburu, Silvia Inés Cazorla, Emilio Luis Malchiodi

**Affiliations:** ^1^Cátedra de Inmunología and Instituto de Estudios de la Inmunidad Humoral Prof. Ricardo A. Margni (IDEHU, UBA-CONICET), Facultad de Farmacia y Bioquímica, Universidad de Buenos Aires, Buenos Aires, Argentina; ^2^Instituto de Microbiología y Parasitología Médica (IMPaM, UBA-CONICET), Departamento de Microbiología Parasitología e Inmunología, Facultad de Medicina, Universidad de Buenos Aires, Buenos Aires, Argentina; ^3^Instituto de Fisiopatología Cardiovascular, Departamento de Patología, Facultad de Medicina, Universidad de Buenos Aires, Buenos Aires, Argentina; ^4^Laboratorio de Inmunología, Centro de Referencia Para Lactobacilos (CERELA-CONICET), Tucumán, Argentina

**Keywords:** Chagas disease, *Trypanosoma cruzi*, cruzipain, chagasin, GM-CSF, therapeutic vaccine

## Abstract

Chagas disease caused by the protozoan parasite *Trypanosoma cruzi* is endemic in 21 Latin American countries and the southern United States and now is spreading into several other countries due to migration. Despite the efforts to control the vector throughout the Americas, currently, there are almost seven million infected people worldwide, causing ~10,000 deaths per year, and 70 million people at risk to acquire the infection. Chagas disease treatment is restricted only to two parasiticidal drugs, benznidazole and nifurtimox, which are effective during the acute and early infections but have not been found to be as effective in chronic infection. No prophylactic or therapeutic vaccine for human use has been communicated at this moment. Here, we evaluate in a mouse model a therapeutic DNA vaccine combining Cruzipain (Cz), a *T. cruzi* cysteine protease that proved to be protective in several settings, and Chagasin (Chg), which is the natural Cz inhibitor. The DNAs of both antigens, as well as a plasmid encoding GM-CSF as adjuvant, were orally administrated and delivered by an attenuated *Salmonella* strain to treat mice during the acute phase of *T. cruzi* infection. The bicomponent vaccine based on *Salmonella* carrying Cz and Chg (SChg+SCz) was able to improve the protection obtained by each antigen as monocomponent therapeutic vaccine and significantly increased the titers of antigen- and parasite-specific antibodies. More importantly, the bicomponent vaccine triggered a robust cellular response with interferon gamma (IFN-γ) secretion that rapidly reduced the parasitemia during the acute phase and decreased the tissue damage in the chronic stage of the infection, suggesting it could be an effective tool to ameliorate the pathology associated to Chagas disease.

## Introduction

Chagas disease, caused by the protozoan parasite *Trypanosoma cruzi*, is endemic in 21 Latin American countries and the southern United States and now is spreading, due to migration, into several other countries (1, 2). Despite the efforts of the World Health Organization and Pan American Health Organization (3) to control the vector throughout the Americas, currently, there are almost seven million infected people worldwide, causing ~10,000 deaths per year by complication linked to Chagas disease, and 70 million people at risk to acquire the infection (4).

Although vector control and screening tests before blood transfusions and organ transplantation have successfully achieved substantial reduction in the number of new acute cases in endemic areas (5), complete control of *T. cruzi* is currently unaffordable. Despite vector control programs effectively reducing the level of infestation, when they are not sustained over time, reinfection of treated households reaches levels like the initial one (6). This was mainly seen in rural areas, when constructions are poor or in peridomestic areas with domestic animals (7). Several concerns remain a challenge, namely, disease globalization due to migration (1, 8); the existence of alternative routes of transmission, such as mother-to-child or oral transmission through contaminated food or beverages (9); and the high variability in drug sensitivity across multiple strains of *T. cruzi* (10).

Despite the fact that 100 years has passed since the discovery of Chagas disease, the treatment is restricted only to two parasiticidal drugs, benznidazole, and nifurtimox, developed in the 1970s and 1960s, respectively. The long-course treatments with these therapeutics ensure efficacy during the early stage of the infection (11); however, they have poor success when administered during the chronic phase of the infection. Moreover, they have severe adverse effects, causing up to 40% of dropout of the treatment and resistance issues being described (12, 13). In this context, safer and more effective drugs or therapeutic vaccines are urgently needed (14, 15).

*T. cruzi* contains a major cysteine proteinase, Cruzipain (Cz), which is expressed by all developmental forms and strains of the parasite; it is secreted and can also be found in the parasite membrane. Parasite virulence and morphogenesis depend on the endogenous activity of the lysosomal Cz (16). In addition, it has been proved that Cz is essential for amastigote replication and plays a crucial role in host–parasite interactions (17). Cz stimulates potent humoral and cellular immune responses during infection (18). Additionally, Cz has been reported as an efficient prophylactic vaccine as both protein and DNA vaccine, in different administration routes and coupled with several adjuvants (19–24). In addition, we had encouraging results using a therapeutic Cz DNA-based vaccine that has been able to decrease parasitemia, inflammatory cell infiltrate, and tissue damage in murine models of *T. cruzi* infection (25).

Cysteine protease inhibitors have been explored as novel anti-*T. cruzi* therapeutic strategies (26–28). Chagasin (Chg), a *T. cruzi* natural protein, is a tight-binding inhibitor of papain-like cysteine proteases. Chg regulates the endogenous activity of Cz, finely modulating proteolytic functions essential for parasite differentiation and invasion to mammalian cells, compromising the virulence and morphogenesis of the parasite. Detailed structural studies showed that Chg is associated with the native cysteine protease in the Golgi compartment (29). However, the proportion of unbound Cz is higher, so only a small amount is found to form the Cz–Chg complex. Actually, this association may be related to the prevention of Cz autocatalysis. It also has been reported that trypomastigotes overexpressing Chg are less infective than wild-type parasites (29). Moreover, Chg is expressed in all developmental stages of *T. cruzi* and can be localized in the flagellar pocket and cytoplasmic vesicles of trypomastigotes and to the cell surface of amastigotes, being an antigen recognized by sera from chronic *T. cruzi*-infected patients (30). The endogenous regulation of Cz by Chg influences important aspects of *T. cruzi* biology, such as morphogenesis, sensitivity to synthetic inhibitors, and infectivity of the parasite (30). These points signal Chg as a relevant antigen to include in a prophylactic or therapeutic vaccine against *T. cruzi* infection, not yet exhaustively investigated.

Based on these backgrounds, we evaluate here a combined strategy with the DNA of both Cz and Chg as therapeutic vaccine, looking for a robust and balanced immune response effective in decreasing blood and tissue parasites and also the characteristic tissue damage of *T. cruzi* infection.

## Materials and Methods

### Parasites

Blood *T. cruzi* trypomastigotes of the RA strain (discrete typing unit (DTU) VI) were isolated from acutely infected mice at the parasitemia peak. Parasite passages were performed weekly in 21-day-old CF1 mice. After 15 days post infection (dpi), parasites were obtained by centrifugation (10,000 × *g*, 30 min) from heparinized blood. Epimastigotes of the same parasite strain were grown in liver infusion tryptose (LIT) medium at 27°C to the exponential phase of growth and centrifuged at 3,000 × *g* for 15 min as previously described (31).

To obtain a soluble antigenic fraction, epimastigotes of *T. cruzi* (RA strain) were centrifuged for 15 min at 5,000 × *g*. The pellet was resuspended in 0.25 M sucrose and 5 mM KCl containing protease inhibitors (2 μM PMSF, 5 μM leupeptin, 5 μM pepstatin, and 5 μM E-64; Sigma, St. Louis, MO), and parasites were lysed by three cycles of freezing and thawing. The homogenate was centrifuged at 105,000 × *g* for 1 h at 4°C, and the supernatant containing *T. cruzi*-soluble antigens was called fraction 105 (F105). The presence of different protein bands in the complex antigen was verified by 10% SDS-PAGE, and F105 was aliquoted and conserved at −20°C.

### Antigens

Purified *T. cruzi* epimastigote DNA was used as a template for the amplification of Cz and Chg. Cz gene was cloned as described by Cazorla et al. (19). Chg amplification was performed using the following primers: Fw 5′-gtcatgcatatgggtgcttgtgggtcgaag-3′ and Rev 5′-gtcatggaattctcagtggtggtggtggtggtgcgcgctctccggcacgttg-3′. The PCR was performed at an annealing temperature of 56°C using a Platinum Taq DNA Polymerase (Invitrogen, Carlsbad, CA). The 333-bp fragment was purified from a 1% agarose gel.

The amplified fragment was digested with the *Nde*I and *Hind*III restriction enzymes, inserted into a pET23a plasmid and transformed in *Escherichia coli* XL2-Blue. The presence of the insert was confirmed by sequence analysis, and the recombinant plasmid was used to transform BL21 cells. Protein expression was induced with 1 mM IPTG ON at 20°C.

rChg was purified under native conditions with growing concentrations of imidazole using a Ni^+2^-nitrilotriacetic acid–Sepharose matrix. After dialysis in PBS–glycerol 10%, the purity and identity of Chg were analyzed. Endotoxins were removed by a polymyxin B–agarose column (Sigma, St. Louis, MO). Endotoxin levels in the final protein preparations were <100 units/mg, as determined using a *Limulus* amebocyte lysate analysis kit (Whittaker Bioproducts, Walkersville, MD).

The catalytic activity of cysteine proteases was measured in a continuous test using the fluorogenic substrate, Z-Phe-Arg-AMC (Bachem). The fluorescence reading of 7-aminomethyl coumarin (AMC) released was measured with a Victor3 fluorometer (PerkinElmer) every 10 seg (λ_excitation_ = 355 nm/λ_emission_ = 460 nm). Increasing concentrations of Chg (0.01–10 μg) were evaluated against epimastigote lysates (5 μM) and the N-terminal domain of Cz (NtCz, 6 μg) previously cloned and expressed (20). The synthetic inhibitor E-64 (10 μM) was used as positive control.

Chg gene for DNA vaccination was cloned in the eukaryotic plasmid pcDNA3.1(+), using the primers fw: 5′-tgatgttgaattcgtaatgtcccacaaggtgacg-3′ and rev: 5′-gtcatgggatcctcagtttgccttgagatatacagtg-3′ that contain the cutoff sites for restriction enzymes, the Kozak sequence, the ATG code for proper transcription, and the TCA termination code. The digested fragment, with the enzymes *Eco*RI and *Bam*HI, was ligated to the plasmid pcDNA3.1(+) (Invitrogen, Life Technologies). Positive transformed *E. coli* DH-5α clones were selected on plates with LB–ampicillin medium. In purified DNA (QIAGEN kit), the presence of the insert of interest was confirmed by digestion with the corresponding restriction enzymes and subsequent sequencing.

Additionally, the Chg-pcDNA3.1 plasmid was used for transforming electrocompetent bacteria *Salmonella enterica* serovar Typhimurium aroA SL7207, which were used as a DNA delivery system as described previously (32).

### Animals

C3H/HeN mice were maintained in the animal facilities of the Instituto de Microbiología y Parasitología Médica (IMPaM, UBA-CONICET) under standard conditions following the Review Board of Ethics of the School of Medicine, UBA, Argentina (Resol. CD #3721/2014) and the guidelines established by the National Research Council (33). Animal experiments were approved by the Review Board of Ethics of the School of Pharmacy and Biochemistry (UBA, Argentina) and conducted in accordance with the National Institutes of Health guide for the care and use of laboratory animals (NIH Publications No. 8023, 2011). Animal sample size was estimated by a power-based method (34) and following the guidelines established by the National Research Council PREPARE guidelines in the design of experiments (35).

### Challenge and Therapy

Immunotherapeutic studies were performed on 6–8-week-old female C3H/HeN mice. Groups of five mice infected with 50 (sublethal dose) or 1,000 (lethal dose) blood trypomastigotes of the RA strain were treated by the oral administration of 10^9^ CFU/dose/mouse of aroA-attenuated *Salmonella* transformed with the different plasmids encoding the antigens or the adjuvant (32). The groups are as follows: (I) **SControl**, attenuated *Salmonella* as carriers of granulocyte-macrophage colony-stimulating factor (GM-CSF) DNA (SGM-CSF); (II) **SChg**. two attenuated *Salmonella*, one transformed with Chg DNA and the other with GM-CSF DNA; (III) **SCz**, one *Salmonella* with the Cz gene and SGM-CSF; and (IV) **SChg+SCz**, *Salmonella* transporting the Chg, Cz, and GM-CSF plasmids.

Acute treatments consisted of three doses of the DNA transported by *Salmonella* on 0, 10, and 20 dpi. Chronic treatment was administered on 100, 110, and 120 dpi. Blood and tissue samples from the mice were taken 100 days after the last immunization.

### Treatment Efficacy

#### Parasitemia and Weight Loss

After challenge with trypomastigotes of *T. cruzi*, blood parasites were counted every 2 days, until the end of the acute phase of infection, as previously described (25). Survival and weight of the animals were recorded daily until they were sacrificed.

#### Specific Immune Response Analyses

Determination of specific antibodies elicited by the vaccines was performed by an indirect ELISA against a complex mixture of soluble *T. cruzi* antigens called F105 (10 μg/ml) or against rCz or rChg (2 μg/ml). Titers were calculated as the inverse of the highest dilution in which the optical density is higher than 0.1.

A delayed-hypersensitivity test (DTH) was performed on day 100 post treatment by measuring the thickness of the footpads prior and 48 h post inoculation of 5 μg of rCz or rChg.

Spleen cell proliferation assays were performed at the end of the experiments in the presence of rCz or rChg (10 μg/ml), F105 (100 μg/ml), Con A (5 μg/ml), or medium alone (control) by triplicate. Eighteen hours before the harvest, 1 μCi per well of thymidine (H)^3^ (Amersham) was added. Proliferative response was expressed as the difference between cpm values obtained from stimulated and nonstimulated cultures.

Splenocyte stimulation by the different antigens was also measured as the intracellular production of interferon gamma (IFN-γ) in a flow cytometry assay. In the last 12 h of incubation, brefeldin A was added to the cultures. Cells were fixed at room temperature (RT) with PFA 2%, permeabilized in 0.5% saponin, and stained using anti-IFN-γ (e-Bioscience conjugated antibody) in accordance with the manufacturer's instructions. Stained cells were passed through the BD FACS II flow cytometer, and data were analyzed with Flow JoX software 10.0.7.

#### Muscle Injury

A hundred days after the last immunization (120 dpi), muscle damage was assessed by measuring the serum activity of creatine kinase (CK and CK-MB), following the instructions of the manufacturer (Wiener Lab).

Histological analyses were also performed at the same time, for which the mice were euthanized, and the corresponding necropsy was performed. The section of muscle (quadriceps) and the complete cardiovascular block were removed. Both ventricles and atria were dissected, and the left ventricle (LV) was cut from the apex to base, fixed in phosphate-buffered 10% formaldehyde at pH 7.2, and embedded in paraffin wax.

Tissues were sectioned into multiple, consecutive 5-μm cross sections with a Reichert–Jung micrometer (Nußloch, Germany) for light microscopic observation as previously described (21). For each heart and skeletal muscle, 20 random microscopic fields were counted at 400 × magnification. Sections were evaluated under blind conditions. The lesion severity was assessed based on semiquantitative criteria according to the inflammation index: (1) isolated foci; (2) multiple nonconfluent foci; (3) multiple confluent foci; and (4) multiple diffuse foci, as previously described (36). Regarding the heart, histological examinations of the LV, right ventricle, and septum were conducted. The presence of fibrosis in the heart sections was evaluated with Masson's trichrome stain without considering the perivascular collagen for each of the groups.

In addition, on the day of euthanasia, 100 mg of skeletal and cardiac muscles was extracted to quantify the parasite burden in each tissue, as described (37). The DNA extraction was made by a phenol–chloroform–isoamyl alcohol mixture (25:24:1 v/v, Quick-DNA, Kalium Technologies). The template for DNA amplification was adjusted to 25 ng/μl. Specific *T. cruzi* primers were used for the DNA amplification (Pr: 5′-AAGCGGATAGTTCAGGG-3′, Pf: 5′-GGCGGATCGTTTTCGAG-3′). As for the normalized gene, samples were also amplified with mouse TNF-α primers (Pf: 5′-CCCTCTCATCAGTTCTATGGCCCA-3′, Pr: 5′-CAGCAAGCATCTATGCACTTAGACCCC-3′). PCR was performed using HOT FIRE Pol Eva Green qPCR Mix Plus (Solis Biodyne). The standard was performed by mixing 500 mg of noninfected mouse muscle with 1 × 10^8^ *T. cruzi* epimastigotes. After the extraction, the DNA was adjusted to 25 ng/μl. For the curve, 10-fold serial dilution of the standard was made using noninfected mouse muscle DNA (25 ng/μl) as diluent. Parasite burden was expressed as parasite equivalent per 50 ng of total DNA.

### Statistical Analysis

Statistical analyses were carried out with GraphPad Prism 6.0 software (San Diego, CA, USA) using one-way analysis of variance (ANOVA) for proliferation, antibody, enzyme activity assays, and parasitemia. Normality was verified using the Shapiro–Wilks test. The specific posttest was indicated in each assay. The log-rank test was used for survival curves, using the Prism 6.0 program (GraphPad, San Diego, CA). The statistical analyses were referred to the control group of each experiment. Values of *p* < 0.05 were considered significant.

## Results

### Cloning and Characterization of Chg

**Figure 1:**
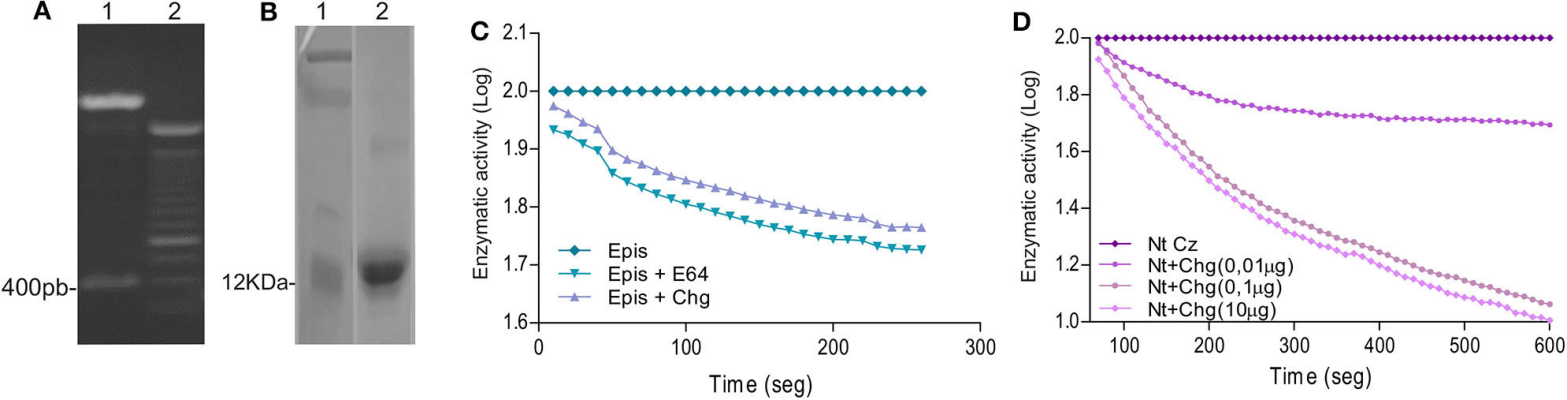
Cloning and characterization of Chg. **(A)** Digestion of pET23a^+^-Chg construction with the *Nde*I and *Hind*III restriction enzymes released the 400-bp Chg insert. **(B)** SDS-PAGE analysis of rChg expression in *E. coli* BL21 and purification under native condition. Lane 1: MW marker; Lane 2: IMAC Ni-NTA purified rChg (12 kDa) stained with Coomassie Brilliant Blue. **(C)** Relative enzymatic activity of *T. cruzi* epimastigote lysate (5 μM) in the presence of rChg (10 μM) or synthetic inhibitor E-64 (10 μM) on the fluorogenic substrate Z-Gly-Pro-AMC (6 μg) measured as relative fluorescent units (RFUs) and expressed as the log of the percentage of the fluorescence units as a function of time. **(D)** Cysteine protease activity of the rNtCz in the absence or presence of increasing concentrations of rChg (0.01–10 μg).

In order to analyze the humoral and cellular-specific response after the therapeutic treatment, Chg and Cz were produced as recombinant proteins. Cz was previously cloned by Cazorla et al. (19). Chg gene was amplified from epimastigote gDNA and cloned in pET23a^+^ plasmid. The obtained pET23a^+^ Chg vector was digested with *Nde*I and *Hind*III restriction enzymes, releasing a 400-bp fragment visualized on a 1% agarose gel (Figure 1A). After sequence confirmation, this vector was used to transform *E. coli* BL21. Chg was obtained as a soluble protein and purified under native conditions; a 12-kDa band in SDS-PAGE was observed (Figure 1B).

To analyze whether rChg maintains the capacity to inhibit cysteine protease activity of the natural Chg, we analyzed the inhibition of enzymatic activity on the fluorogenic substrate Z-Phe-Arg-AMC. High contents of cysteine proteases, including Cz, are present in the *T. cruzi* replicative epimastigote stage, and we observed that rChg was able to inhibit the activity from lysed epimastigotes with a similar slope to the commercial synthetic inhibitor (E-64) used as positive control (Figure 1C). To analyze rChg-specific inhibitory capacity on Cz, the catalytic domain of Cz (rNtCz) was assayed in the presence or absence of rChg, and a well-defined concentration-dependent inhibition was observed (Figure 1D).

### SChg+SCz as a Therapeutic Vaccine Against a Lethal *T. cruzi* Infection

**Figure 2:**
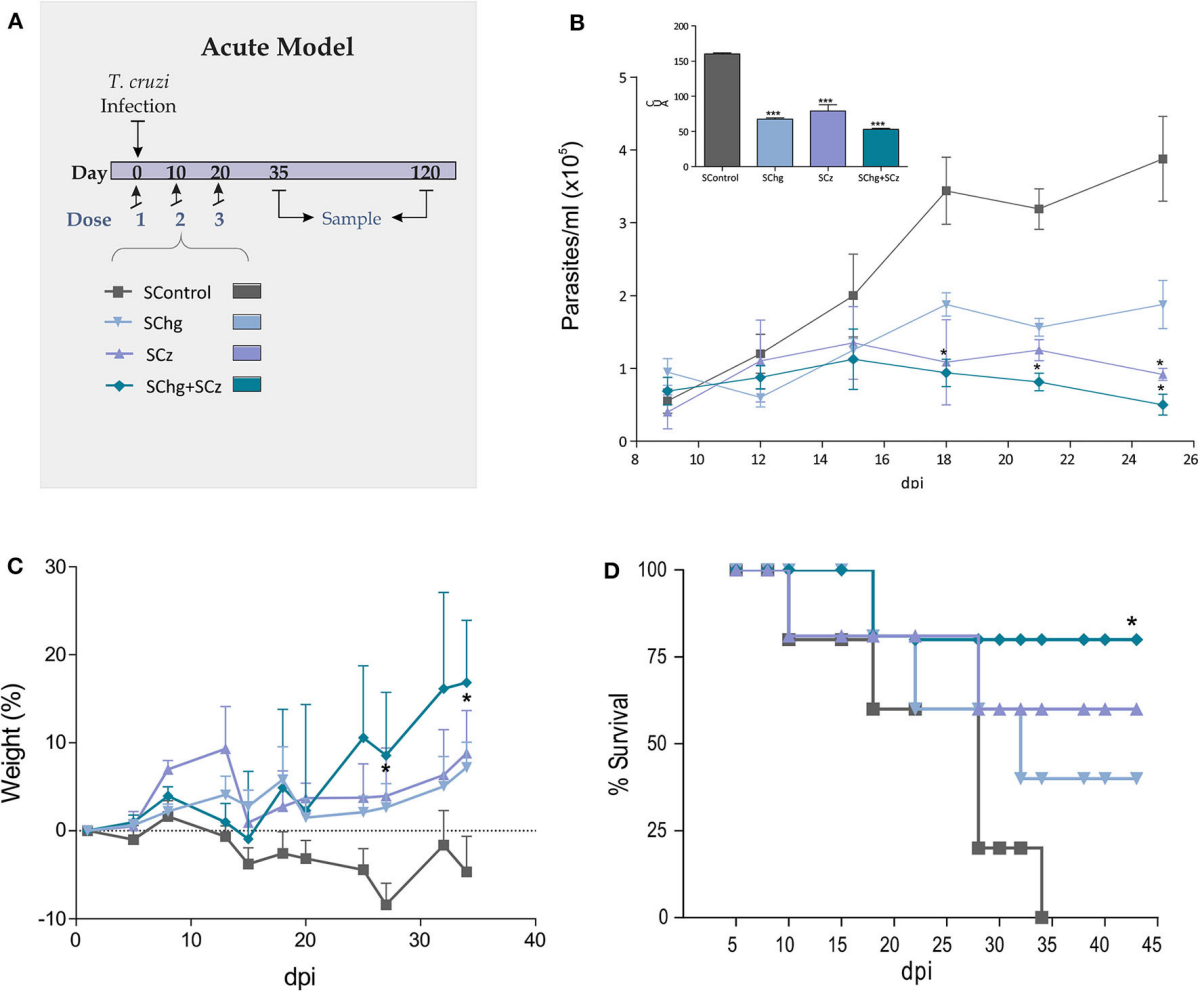
Protection against *T. cruzi* challenge. **(A)** Acute model of infection: C3H/HeN mice infected with *T. cruzi* were treated during the acute phase of infection at 0, 10, and 20 dpi, with three doses of *Salmonella* transporting Cz and/or Chg together with GM-CSF (SCz, SChg, and SChg+SCz, respectively). **(B)** Parasitemia curve after a lethal challenge (insert) AUC of parasitemia. **(C)** Change in body weight of immune-treated mice. **(D)** Survival rate curve evaluated every 2–3 days. These results are representative of at least three independent experiments. One-way ANOVA plus Dunnett's posttest or Tukey's posttest and log-rank test for survival curves were used. Results are expressed as mean ± standard error (*n* = 5 per group), *p < 0.05, ***p < 0.001.

To improve the therapeutic effect afforded by Cz DNA (25), we evaluated the coadministration with Chg DNA. Mice lethally infected with *T. cruzi* (1,000 RA trypomastigotes/mouse) were treated with three doses of *Salmonella* delivering the DNA encoding for GM-CSF (SGM-CSF) as adjuvant and Chg (SChg), Cz (SCz), or a combination of both (SChg+SCz). The control group was treated with *Salmonella* carrying only the DNA of GM-CSF (SControl) (Figure 2A).

During the acute phase of the infection, all treated mice showed an important decrease of the parasitemia across the infection compared to the control group (Figure 2B). As the box of Figure 2B shows, the area under the curve (AUC) highlights the significant differences between the control group (AUC: 158.60) and treated ones, SChg (AUC: 69.63), SCz (AUC: 87.28), or Chg+SCz (AUC: 54.75) (*p* < 0.005). The reduction in parasitemia was reflected in body weight assessment during this phase, showing substantial differences in all treated mice in comparison to controls (Figure 2C). Thus, control mice weight loss correlated with high parasitemia and 100% of mortality at 34 dpi (Figure 2D). In contrast, mice receiving the therapeutic vaccine based on *Salmonella*s carrying both Cz and Chg DNAs had a survival rate of 75% (^*^p < 0.05), while in mice treated with SCz or SChg, the survival rates were 60% and 40%, respectively (Figure 2D).

### SChg+SCz Therapeutic Vaccine After a Sublethal Infection Enhances *T. cruzi*-Specific Immune Response

**Figure 3:**
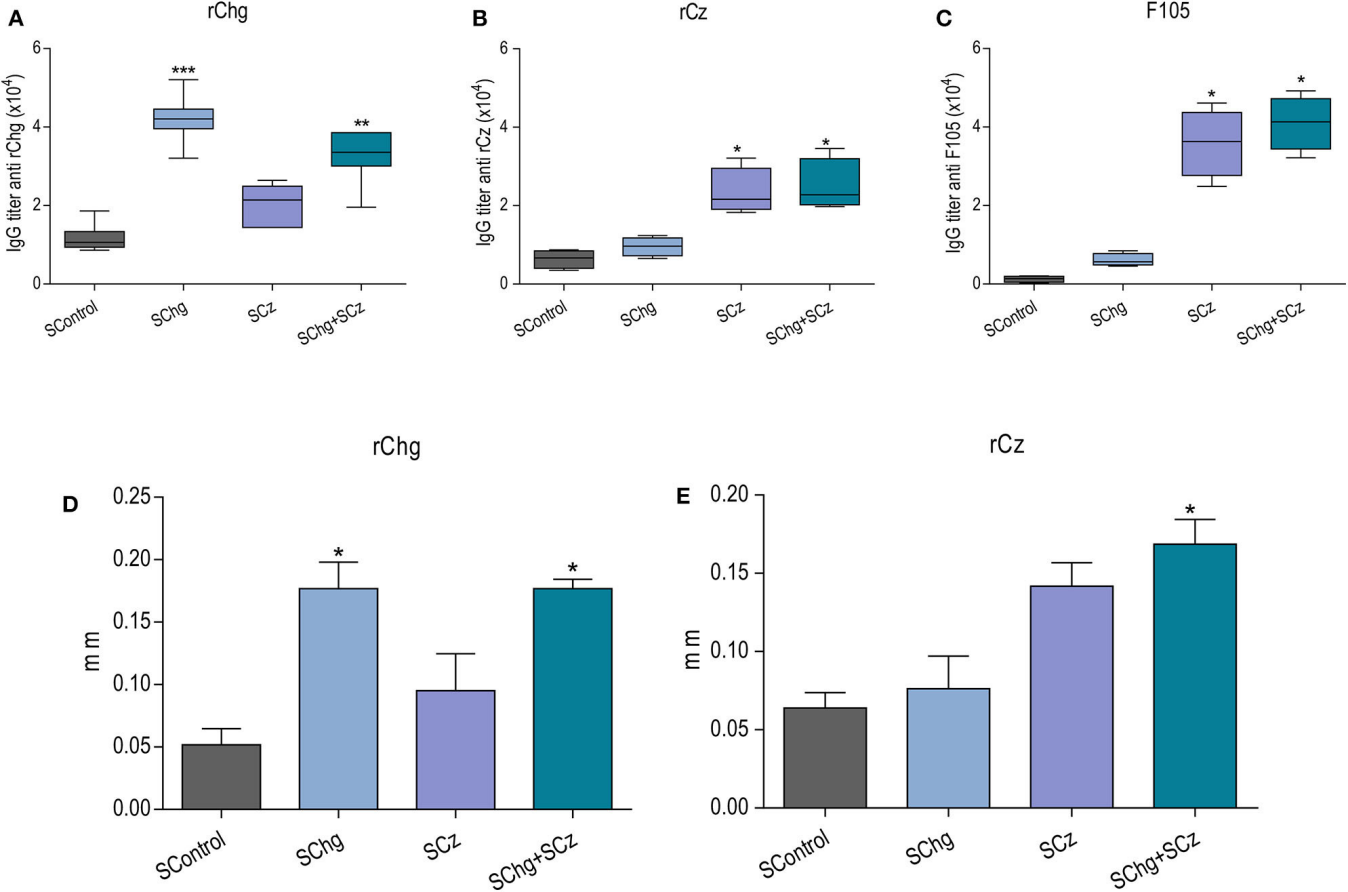
Immune response in mice treated during the acute phase of *T. cruzi* infection. Mice infected with a sublethal dose of *T. cruzi* were treated at 0, 10, and 20 dpi with doses of *Salmonella* carrying the DNA of Chg and/or Cz together with the GM-CSF. Antibody immune response: Total IgG titers of **(A)** anti-rChg, **(B)** anti-rCz, and **(C)** anti-F105 were measured in sera of all groups obtained at 35 dpi. *In vivo* cellular immune response: Footpad thickness was measured before and 48 h after intradermal inoculation of **(D)** rChg or **(E)** rCz. The bars represent the average (*n* = 5 per group) of three representative experiments. *p < 0.05, **p < 0.01, ***p < 0.001.

To study the effect of the immunotherapies on the specific immune response, at the end of the acute phase (35 dpi) or the chronic phase (120 dpi) of the parasite infection, another set of mice was infected with a sublethal dose (50 RA trypomastigotes per mouse) and treated as previously describe (Figure 2A). The presence of specific IgG antibodies against immobilized rCz, rChg, and a *T. cruzi* lysate F105 was monitored when the acute phase of infection was overcome, at 35 dpi (Figure 3). At that time, a significant increase in IgG antibodies against rCz and F105 was observed in mice treated with SCz and SChg+SCz (Figures 3B,C), but not in the SChg-treated mice. By contrast, this group had significant rChg-specific IgG titers compared to controls (Figure 3A). Notably, infected and vaccinated mice developed a strong humoral immune response to the immunizing antigen in spite of the ongoing *T. cruzi* infection. When the immune response was analyzed against the natural antigens present in the parasites (F105), a significant antibody titer was observed only in groups that received Cz in the formulation. Just the group that received both antigens (SChg+SCz) had a significant antibody response against all parasite antigens (Figures 3A–C). The lack of reactivity against F105 in sera from the SChg group could be related with the fact that Chg is much less represented in F105 than Cz, which is a major antigen.

Furthermore, the specific cellular immune response was analyzed *in vivo* by a delayed hypersensitivity test (DTH). As expected, at the end of the acute phase, the groups treated with the different strategies responded in a significant way to the stimulation with their corresponding antigen, rCz or rChg (Figures 3D,E).

### The Immune Response Elicited by the SChg+SCz Therapeutic Vaccine Is Long Lasting and Persists During the Chronic *T. cruzi* Infection

**Figure 4:**
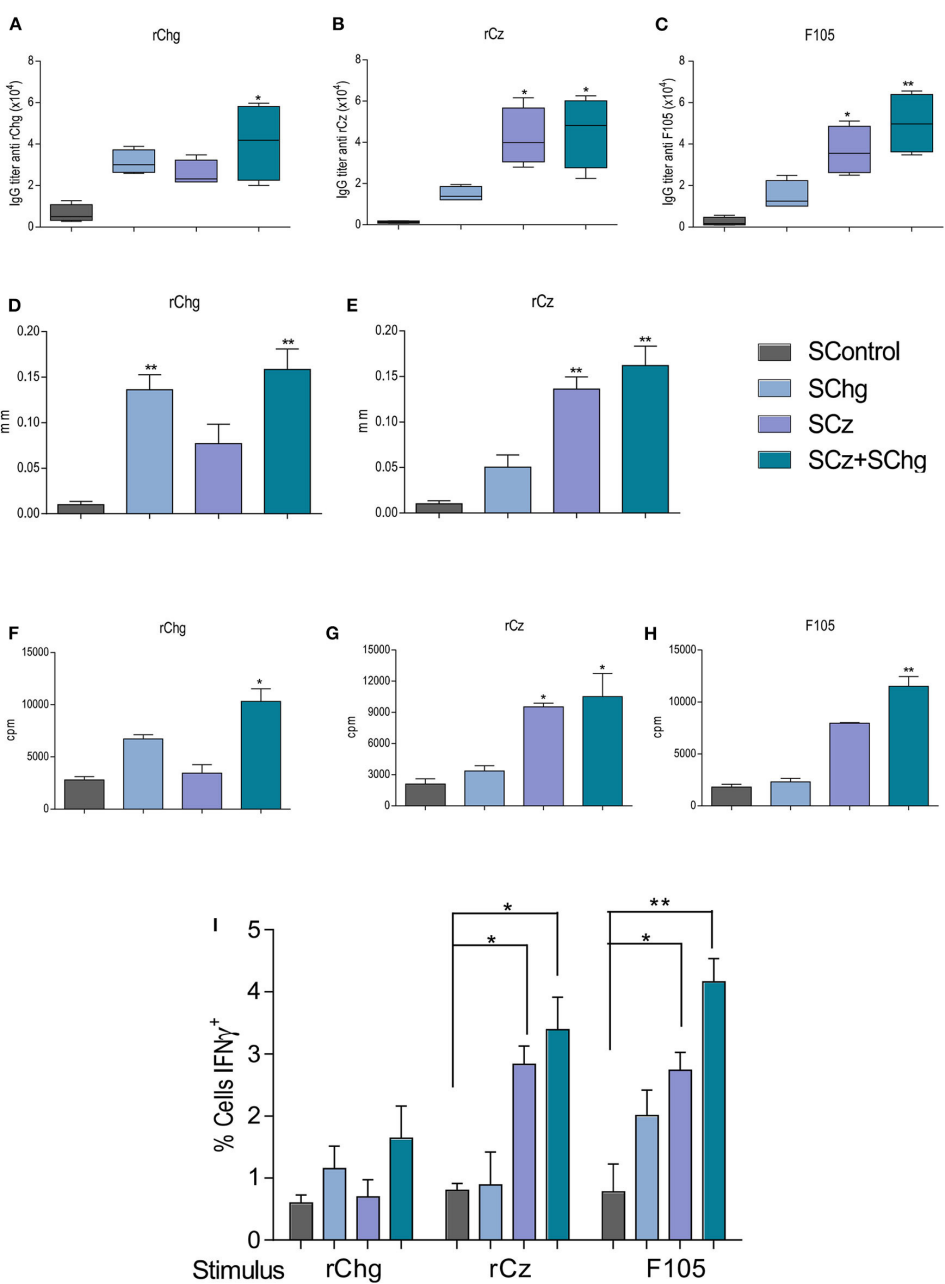
Immune response elicited by the therapeutic vaccine at 120 dpi, in mice treated during the acute phase of *T. cruzi* infection. Mice infected with a sublethal dose of *T. cruzi* were treated with three doses of *Salmonella* at 0, 10, and 20 dpi (*n* = 5 per group), and the immune response generated was analyzed at 120 dpi. The IgG titers **(A)** anti-rChg, **(B)** anti-rCz, and **(C)** anti-F105 were measured in sera at 120 dpi. Cellular response: The *in vivo* DTH assay was performed by measuring the plantar footpad before and 48 h after intradermal inoculation of **(D)** rChg or **(E)** rCz; results are expressed as the differences in thickness. Splenocytes from mice sacrificed at 120 dpi were restimulated with 10 μg/ml **(F)** rCz, **(G)** rChg, or **(H)** F105 for 5 days, and thymidine uptake was determined. The results are expressed as the differences between the cpm of stimulated and baseline cells. **(I)** Intracellular production of INF-γ, determined by flow cytometry, after the stimulation of the splenocytes with the different antigens. These results are representative of at least three independent experiments. *p < 0.05, **p < 0.01.

To further characterize the performance of the therapeutic *T. cruzi* vaccines, the elicited immune response was also analyzed during the chronic phase of the infection (120 dpi). We observed that the humoral immune responses against rChg and rCz were slightly increased compared to those observed at the end of the acute phase (35 dpi) for all groups analyzed. This is mainly for SChg+SCz-treated mice which showed significant differences to the control group (Figures 4A–C).

The DTH at this time point showed a significant increase (^*^p < 0.01) of the *in vivo* specific cellular immune response against the antigens that were administered during the therapy (Figures 4D,E), according to what was observed at the end of the acute stage of the infection.

In addition, 100 days after last immunization, we evaluated *ex vivo* the lymphoproliferative response of splenocytes from vaccinated mice upon the restimulation with each antigen: rChg, rCz, or F105. Spleen cells from SCz alone or in combination with SChg significantly proliferate in response to rCz and F105 (Figures 4F–H).

IFN-γ is an inflammatory cytokine crucial for immunity against *T. cruzi*, involved in the control of systematic parasite dissemination (38, 39). At 120 dpi, we observed a significant increase in the percentage of IFN-γ-producing cells in splenocytes from SChg+SCz-treated mice after being stimulated with Cz, Chg, or F105, compared to untreated mice infected with *T. cruzi*. SCz spleen cells responded to Cz or F105 incubation, while SChg only responded to the parasite's lysates (Figure 4I).

### SChg+SCz Therapeutic Vaccine Prevents Tissue Damage of the Chronic Infection

Then, we analyzed if the therapeutic vaccines administered during the acute phase of the parasite infection were able to prevent the tissue damage associated to the chronic phase of *T. cruzi* infection. To this purpose, at day 100 after the last vaccine doses (120 dpi), three parameters were evaluated: serum levels of CK and CK-MB enzymes, histological analysis of tissue inflammation, and parasitism in skeletal and cardiac tissues.

**Figure 5:**
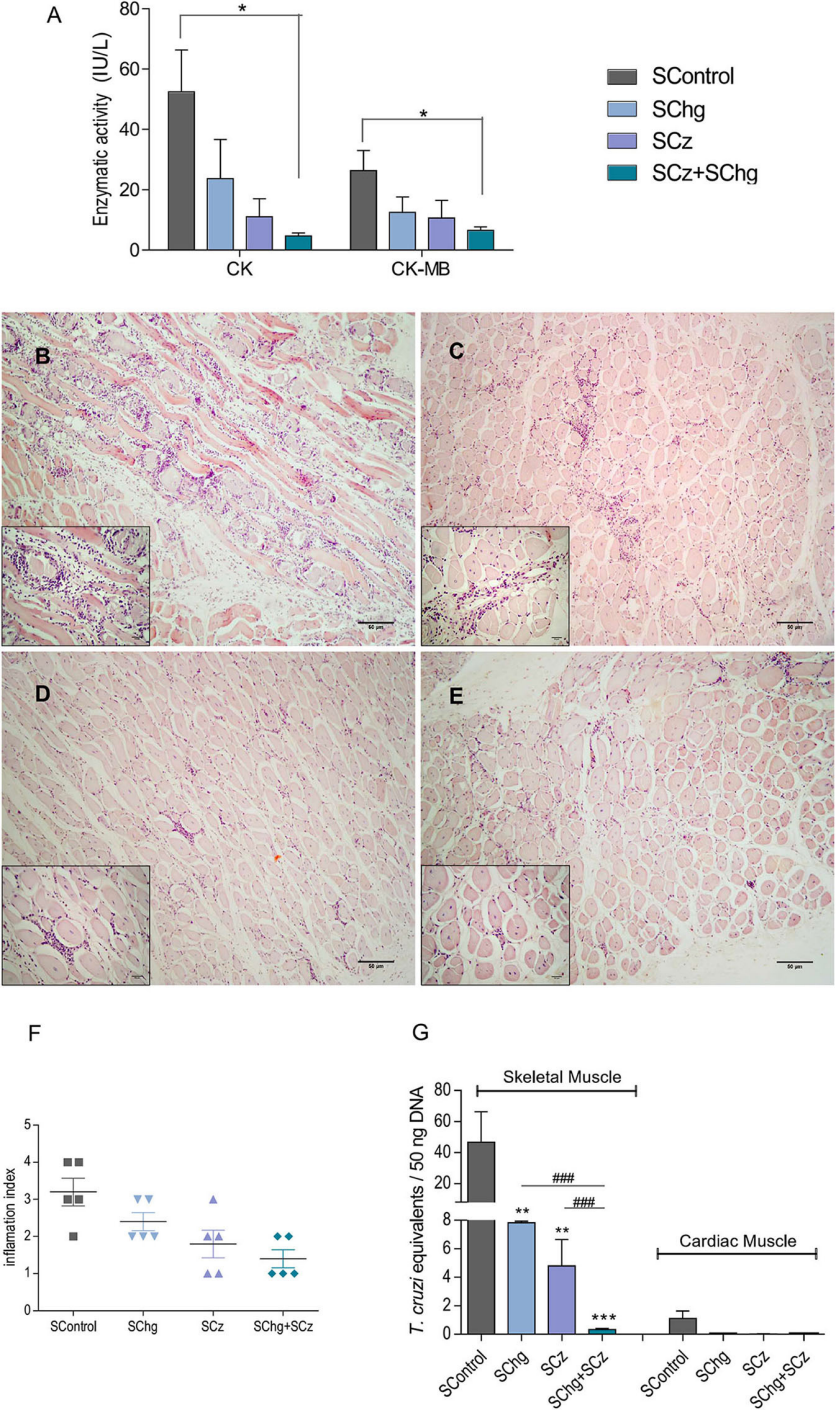
Prevention of tissue damage by vaccine treatment during the acute phase of the parasite infection. **(A)** Enzymatic activity of CK and CK-MB enzymes represented as international units (IU/L). **(B–E)** Representative image of hematoxylin–eosin-stained skeletal muscle samples taken at day 100 after the last immunization; magnification: 40× and the inbox 100×. Animals showed **(B)** multiple confluent foci and necrosis with diffuse distribution (SControl); **(C)** nonconfluent mononuclear inflammatory infiltrate (SChg); **(D)** isolated foci of mononuclear inflammatory infiltrate (SCz); **(E)** few and isolated infiltrates (SChg+SCz). **(F)** Semi-quantified inflammation expressed as inflammation index: (1) isolated foci; (2) multiple nonconfluent foci; (3) inflammatory confluent foci; and (4) multiple diffuse foci (40). **(G)** Parasitism in skeletal and cardiac tissues by qPCR at day 100 after the last immunization. Parasite burden in each tissue was expressed as *T. cruzi* equivalents per 50 ng of total DNA referred to a calibration curve previously constructed containing known concentrations of *T. cruzi* epimastigotes. These results are representative of at least three independent experiments, each one being carried out with five animals per group. *p < 0.05, **p < 0.01, ***p < 0.001; ^*###*^*p* < 0.01.

Serum levels of CK and CK-MB enzymes were assessed as an indicator of muscle injury. As can be seen in Figure 5A, the CK and CK-MB values were lower in all mice that received the therapeutic vaccines compared to nontreated controls. However, these differences were only significant compared to SControl (CK: 52.4 ± 13.9 IU/L; CK-MB: 26.4 ± 6.6 IU/L) in the group that received the *Salmonella* with the combination of both antigen DNAs SChg+SCz (CK: 4.7 ± 1.0 IU/L; CK-MB: 6.6 ± 1.1 IU/L), reflecting a significant reduction in muscle damage.

In addition, histological studies of *T. cruzi* target tissues were performed. We observed low levels of pericardial infiltrates mainly in the right ventricular area without significant differences between treated and nontreated control groups when cardiac tissues were analyzed (results not shown). However, histological sections of skeletal muscle showed necrosis of muscle fibers and numerous multifocal and diffuse inflammatory infiltrates in the control group (Figure 5B). By contrast, all treated groups presented few necrotic zones and isolated nonspecific inflammatory foci (Figures 5C–E). The tissue inflammation was analyzed through a semiquantitative comparative analysis of the different groups, showing that this protective effect was observed mainly in those that received SCz as a monocomponent or bicomponent therapeutic vaccine (Figure 5F).

To determine *T. cruzi* persistence, the parasite load in target tissues was determined by quantitative PCR (qPCR). *T. cruzi* equivalents in cardiac tissues were very low and presented no significant differences in vaccinated animals compared to control mice. However, when parasite loads were analyzed in skeletal muscles, a significant difference was detected between groups (Figure 5G). The control group presented parasite loads between 6 and 10 times higher than those in animals that received the mono-therapeutic vaccine. A striking difference with SControl was observed in animals treated with the combined formulation (SChg+SCz). Importantly, this reduction of parasitic load was more than 100 times. Notably, a significant difference was observed between the groups immunized only with one antigen and the combined treatment (Figure 5G), highlighting the higher efficacy of the SChg+SCz vaccine compared with the mono-therapeutic vaccines.

### Tissue Damage Prevention Is Achieved by SChg+SCz Therapeutic Vaccine Even Administered in the Chronic Phase of *T. cruzi* Infection

**Figure 6:**
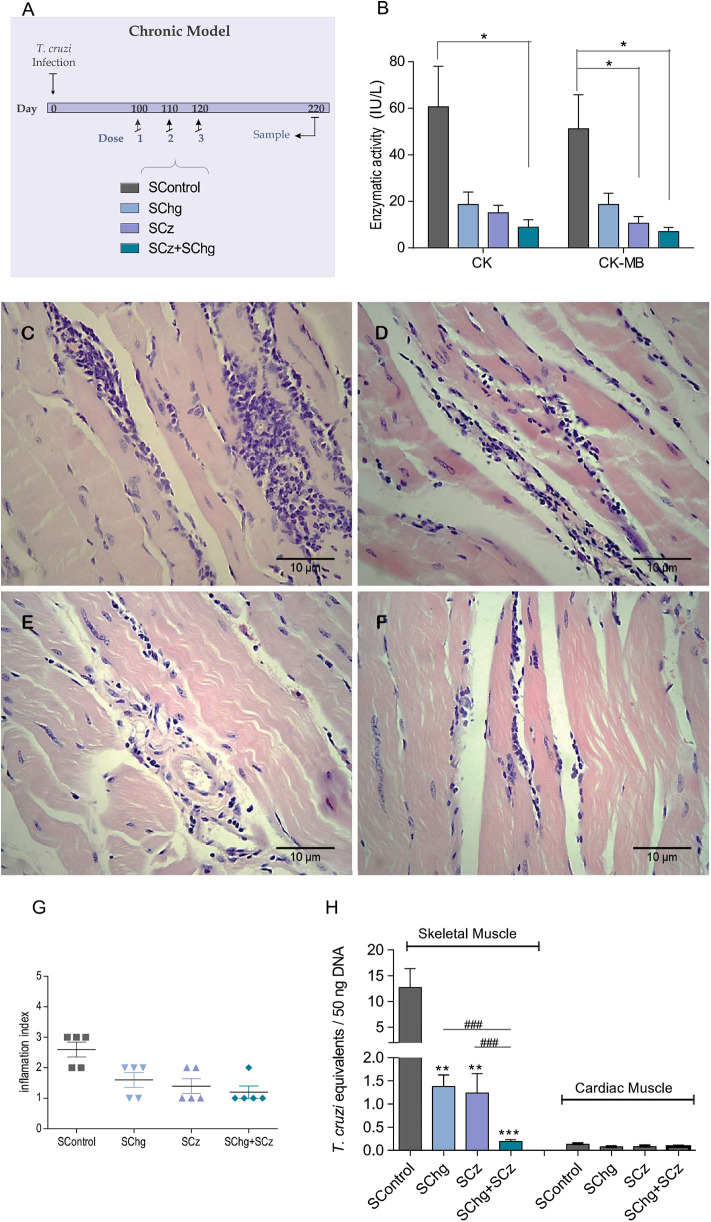
Prevention of *T. cruzi*-associated tissue damage by the vaccines administered at the chronic phase of the parasite infection. **(A)** Scheme of the chronic model of infection: C3H/HeN mice infected with *T. cruzi* received three doses of the SControl, SChg, SCz, or SChg+SCz on 100, 110, and 120 dpi (*n* = 5 per group). **(B)** Enzymatic activity of CK and CK-MB enzymes represented as international units (IU/L). **(C,D)** Histopathological analysis of skeletal tissue samples at 220 dpi showed **(C)** confluent foci of mononuclear inflammatory infiltrate with necrosis of the muscle fibers in SControl; **(D)** isolated foci of mononuclear inflammatory cells with interstitial and perivascular predominance (SChg); **(E)** nonconfluent mononuclear cells surrounding muscle fibers and interstitial edema (SCz); and **(F)** isolated mononuclear cells at the interstitial and perivascular level (SChg+SCz). **(G)** Inflammation semi-quantified and expressed as an index of inflammation: (1) isolated foci; (2) multiple nonconfluent foci; (3) inflammatory confluent foci; and (4) multiple diffuse foci (40). **(H)** Parasite load in skeletal and cardiac tissues determined by qPCR at 220 dpi. Parasite burden in each tissue was expressed as *T. cruzi* equivalents per 50 ng of total DNA. Results were referred to a calibration curve previously constructed containing known concentrations of *T. cruzi* epimastigotes. These results are representative of at least three independent experiments. *p < 0.05, **p < 0.01, ***p < 0.001; ^*###*^*p* < 0.01.

After the promising results observed upon the administration of the therapeutic vaccines during the acute phase, conducted using the acute model described in Figure 2A, we evaluated the performance of the vaccines when administered during the chronic phase of the infection (Figure 6A). *T. cruzi*-infected mice were treated with three doses of the corresponding vaccines on 100, 110, and 120 dpi. At 220 dpi, mice were euthanized, and different parameters were analyzed to assess tissue damage and parasite persistence.

Serum levels of the enzymes CK and CK-MB were significantly lower in SChg+SCz animals (CK: 8.9 ± 3.3 IU/L and CK-MB: 7.0 ± 1.8 IU/L) with respect to control animals (CK: 60.7 ± 17.4 IU/L and CK-MB: 51.2 ± 14.6 IU/L) (Figure 6B). In accordance with these results, the histological analysis of skeletal muscle showed confluent foci of mononuclear inflammatory infiltrate with necrosis of the muscle fibers in SControl group (Figure 6C). By contrast, in mice treated with the therapeutic vaccines, the foci of mononuclear inflammatory cells were isolated with poor or no necrosis of the skeletal fibers (Figures 6D–F). Moreover, animals that received the SChg+SCz vaccine showed isolated inflammatory mononuclear cells with interstitial and perivascular localization. These results are summarized in Figure 6G.

To analyze parasite persistence in target tissues, *T. cruzi* equivalents were determined by qPCR. Again, parasite load in cardiac tissues was very low and did not show differences between groups. However, *T. cruzi* equivalents detected in the skeletal muscle of infected mice treated late during the chronic phase of the infection were significantly lower compared to those in SControl (Figure 6H).

By contrast, serum enzymatic activity of CK and CK-MB and histopathological analysis of tissue and *T. cruzi* equivalents by qPCR in skeletal and cardiac tissues of 220-day-old noninfected mice are shown in Supplementary Figure 1. As expected, noninfected mice were negative by qPCR. CK and CK-MB values in these mice were similar to those in infected and SChg+SCz-vaccinated mice. In noninfected mice, we observed a conserved architecture of both skeletal and cardiac tissues (Supplementary Figure 1), indicating that our treatments were not able to completely avoid skeletal tissue damage. However, the combination of SChg+SCz strongly protected the tissue from damage compared with nontreated mice.

Considering the lack of effective therapeutics during the chronic phase of *T. cruzi* infection, these results suggest that the SChg+SCz-based vaccine would be a promising immunotherapeutic strategy with the potential to decrease the prevalence of Chagas disease.

## Discussion

Therapeutic vaccines for indeterminate and chronic Chagas disease not only would provide health benefits but also result in economic benefits (15). Hence, millions of people are waiting for a development of anti-*T. cruzi* vaccines for prophylactic and therapeutic purposes to stop Chagas disease progression. Even though etiologic treatment based on nifurtimox and benznidazole is undisputed in the acute phase, achieving cure rates between 65 and 80%; trypanocidal treatment in the chronic stages of the disease remains controversial because of significant toxicity and the unproven role in preventing progression to cardiomyopathy (11, 12, 41, 42). What worsens the situation is that just a reduced percentage of infections are detected in the acute phase due to mild symptoms.

In the last few years, a better understanding of the protective immune responses that can effectively arrest *T. cruzi* survival in mammalian host provides the fundamentals for a rational design of a prophylactic and therapeutic vaccine against Chagas disease (22). Anti-*T. cruzi* strategies had been focused on targeting specific metabolic biochemical pathways or parasite-specific enzymes, as well as noninvasive immunization routes (23, 24). Most of these strategies need potent adjuvant to obtain the desirable response to improve vaccine efficacy (43). Recently, DNA vaccines have become an effective tool in a wide variety of preclinical models and have been adopted by several researchers. The intramuscular injection of the plasmid DNA encoding several surface antigens or secreted by *T. cruzi* has been studied as DNA vaccine (5, 44, 45). However, it is known that after intramuscular DNA injection, the amount of DNA available for gene expression is very low, which means that high initial amounts of the plasmid must be applied to obtain satisfactory results (25, 46). The difficulties with naked DNA prompted research into DNA delivery systems using attenuated viruses, bacteria and parasites, liposomes, virosomes, microspheres, nanoparticles, and physical delivery systems, such as electroporation, microinjection, gene gun, tattooing, laser, and ultrasound (38, 47–49).

In our laboratory, we took advantage of attenuated *Salmonella enterica* serovar Typhimurium aroA that can be administered orally and is easily produced, facilitating its implementation in vaccination campaigns. We have previously demonstrated the efficacy of this transport system as prophylactic vaccines to *T. cruzi* infection (19, 21, 24, 50, 51). Based on these promising results, we have evaluated the therapeutic effect of *Salmonella* as a DNA delivery system of Cz upon the coadministration with GM-CSF as adjuvant with good performance (25). With the aim to further increase the therapeutic effect of the vaccine, in the present work, we included Chg, a natural inhibitor of cysteine proteases, as a new immunogen against *T. cruzi* infection. A multicomponent therapy might induce a robust parasite-specific immune response even in late infection, controlling tissue damage and preventing Chagas disease progression.

We first cloned Chg DNA in a prokaryotic expression plasmid and expressed the recombinant protein. The activity analysis demonstrated that rChg is properly folded, interacts with rCz, and is active as a protease inhibitor. Then, we cloned Chg in a eukaryotic expression plasmid and evaluated whether its combination with the plasmid encoding Cz was able to control an ongoing acute and chronic *T. cruzi* infection in mice when transported by *Salmonella* as a DNA delivery system. Interestingly, we found that the bicomponent immunotherapeutic vaccine based on *Salmonella* (SChg+SCz) was able to improve the performance obtained by each antigen as a mono-therapeutic vaccine. The efficacy would be due, at least in part, to the high humoral and cellular responses elicited in SChg+SCz not only to the antigens included in the vaccine (Chg and Cz) but also against the whole parasite as observed when immobilizing the F105 lysate in ELISAs or by the IFN-γ production by spleen cells upon restimulation with the same parasite homogenate. In addition, SChg+SCz-treated mice displayed a significant cellular immune response against both antigens compared to untreated mice or mice that receive a mono-therapeutic vaccine, which could decrease parasite replication inside the host.

Our results are in line with diverse approaches exhibiting the efficacy of several *T. cruzi* antigens (ASP-2, enolase, TSA-1, and Tc24) to control *T. cruzi* infection when they are tested as a therapeutic vaccine during the acute phase of the parasite infection (47, 48, 52–54). Nevertheless, only a few groups evaluated the performance of a therapeutic vaccine when it is administered during the chronic phase of *T. cruzi* infection (38, 43, 55–57). In that regard, the effectiveness of the multicomponent therapeutic vaccine SChg+SCz, when it is orally administered during the chronic phase of the infection, becomes even more relevant. Likewise, like the response observed by a treatment delivered by type 5 recombinant adenoviruses where the IFN-γ-mediated immunity was enhanced (38), treatment of chronically infected mice with DNA of Cz and Chg delivered by *Salmonella* displayed a robust cellular immune response with high levels of IFN-γ secretion upon the restimulation with *T. cruzi* antigens. This is true, particularly, in treatments that include the Cz antigen. Treating mice during the acute phase with the multicomponent vaccine (SChg+SCz) would allow a rapid T-cell response in case of *T. cruzi* reactivation or reinfection and, therefore, a major control of the parasite dissemination to the host tissues. This supports the idea of other authors that IFN-γ is important in mediating an effective immunity against *T. cruzi* (39).

We analyzed the ability of the immunotherapy to prevent or decrease the onset of tissue damage during the chronic stage of parasite infection. Serum activity of the CK enzyme and the cardiac isoform CK-MB, analyzed as highly specific markers of myocardial injury, showed that SChg+SCz-treated mice, independently of the time of administration, presented a mean level 10 times lower than that observed in infected and untreated mice. Importantly, the SChg+SCz immunotherapeutic vaccine administered at the chronic phase of *T. cruzi* infection was able to improve several parameters that were already altered at the start of the treatment. For example, the serum level of CK-MB in infected and nontreated mice at 100 dpi was 26.4 ± 6.6 IU/L (Figure 5B). When immunotherapy was administered at the chronic phase, CK-MB levels were reduced to 7.0 ± 1.8 IU/L at 220 dpi, values similar to those in normal tissues (Supplementary Figure 1A); otherwise, in the absence of treatment, it reached levels of 51.2 ± 14.6 IU/L (Figure 6B). This decrease in tissue damage by chronic immunotherapy, also described by Pereira et al. (38), is really encouraging since current treatment with nifurtimox or benznidazole at the chronic phase of the parasite infection in humans reduces parasite load but does not improve the clinical outcome of Chagas disease (11). As murine and human infections differ in several aspects of the parasite–host relationship, including duration of infection, these results support the evaluation of anti-*T. cruzi* immunotherapy in human clinical trial basis.

Effectively, these results are a clear evidence that SChg+SCz as an oral therapeutic vaccine administered during the acute or chronic stage of infection has potential to delay the progression of Chagas disease and could be an effective tool to mitigate the damage associated to Chagas disease. In addition, despite the fact that tissues of infected-treated mice are not preserved to the same architecture of the organs of noninfected nontreated age-matched mice (Supplementary Figures 1C,D), there were only a few and isolated inflammatory foci. These tissue damage parameters have a clear correspondence with the parasitic load analyzed by qPCR in cardiac and mainly skeletal muscles, since mice immunized with the bicomponent vaccine presented the highest reduction compared with infected control and experimental groups. These results reinforce the hypothesis that the reduction in tissue parasitism highly correlates with damage in target tissues. Regarding the performance of SChg+SCz, we demonstrated its immunogenicity, being able to prime a more robust immune response. Moreover, with this multivaccination strategy, we were able to improve the efficacy conferred with the administration of the DNA antigens individually. It is also encouraging since the hopeful SChg+SCz treatment could be administered when an acute *T. cruzi* infection is suspected or confirmed. Interestingly, considering that the diagnosis of a *T. cruzi* infection is usually achieved during the chronic phase of the parasite infection, it is of great importance that a tissue protective effect is displayed by the multicomponent therapeutic vaccine when administered on the chronic stage of infection. The major advantage of this strategy is that it relies on the induction of multiple effector mechanisms against the pathogen that may have higher efficacy than conventional treatments to face the asymptomatic and chronic phase of *T. cruzi* infection. Considering animal model limitations and that this pathology is a dynamic process accounting for organ-specific damage, it would be interesting to analyze the efficacy of the treatment using other mouse models and parasite strains that affect different tissues, such as that made by Francisco et al. (58), in further communications.

A therapeutic vaccine for Chagas disease may improve or even replace the treatment with current drugs which have several side effects and require long-term use that frequently leads to therapeutic withdrawal. The oral therapeutic vaccine SChg+SCz would also be an interesting weapon in combined therapies with parasiticidal drugs, as have been successfully reported in different pathologies (59, 60) and reviewed by Bivona (22). With the active replication of the parasite during the acute *T. cruzi* infection being kept in mind, it would be interesting to evaluate if combined therapy administered during the acute phase of the infection could have a synergism with the host immune response and the drug activity to achieve parasite clearance. This immunotherapeutic vaccine not only would allow targeting different humoral and cellular elements as well metabolic pathways but also would be able to reduce doses and drug exposure periods, thus contributing to the decrease in toxic effects and minimization of the emergence of drug resistance. Not to be underestimated, as has been recently reviewed by Rios et al. (61) and proposed by Dumonteil et al. (62), therapeutic vaccines could be an excellent tool to avoid future congenital transmission, which is estimated at 5% in Latin America, preventing the risk of pregnancy complications like preterm rupture of membranes and preterm delivery. Although available drugs are not approved for use during pregnancy, several studies suggest that reducing maternal parasitemia before conception reduces the risk of congenital transmission (63–65), and it was recently estimated that a therapeutic vaccination of Chagas-positive pregnant women would be cost-effective and cost saving (66).

With the bicomponent therapeutic vaccine SChg+SCz, we were able to improve the efficacy conferred by the administration of the DNA antigens individually. This treatment increased the survival of mice upon a lethal *T. cruzi* challenge and successfully decreased tissue damage associated to *T. cruzi* infection. These results indicate that SChg+SCz could be a promising strategy in the search of an anti-Chagas therapy to be administered in either the acute or chronic phase of the infection.

## Data Availability Statement

All datasets presented in this study are included in the article/ Supplementary Material.

## Ethics Statement

The animal study was reviewed and approved by Review Board of Ethics of the School of Pharmacy and Biochemistry (UBA, Argentina) and conducted in accordance with the National Institutes of Health guide for the care and use of Laboratory animals (NIH Publications No. 8023, 2011).

## Author Contributions

NC, AB, AS, SC, and EM: conceived and designed the experiments. NC, AB, AS, ST, CM, and AC: performed the experiments. NC, AB, AS, CM, SC, and EM: analyzed the data. EM: contributed reagents, materials and analysis tools. NC, SC, and EM: wrote the paper. All authors revised and approved the manuscript.

## Conflict of Interest

The authors declare that the research was conducted in the absence of any commercial or financial relationships that could be construed as a potential conflict of interest.

## Supplementary Material

Supplementary Figure 1 Normal tissue analyses at 220 days old animals. **(A)** Serum enzymatic activity of CK and CK-MB represented as International Units (IU/L); **(B)** *T. cruzi* equivalents by qPCR in naïve tissue; Histopathological analysis of non-infected mice, representative image of hematoxylin-eosin stained: **(C)** Cardiac Tissue; **(D)** skeletal muscle. Animals showed a conserved architecture of both tissues. Magnification: 40x.
