# Supplementary figures and images for: Cruzipain and Its Physiological Inhibitor, Chagasin, as a DNA-Based Therapeutic Vaccine Against Trypanosoma cruzi
Source: Front Immunol. 2020 Oct 9;11:565142. doi: 10.3389/fimmu.2020.565142 (PMC7583359; doi:10.3389/fimmu.2020.565142)

A

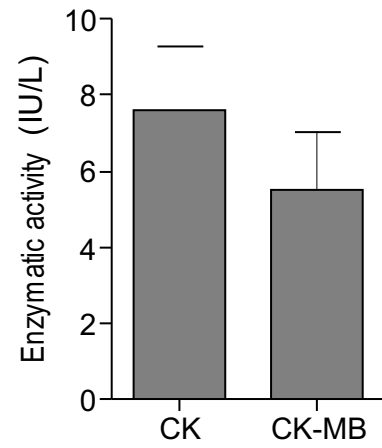

B

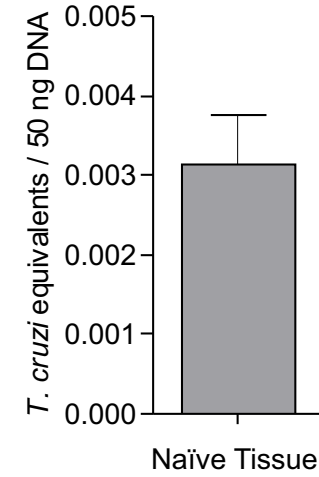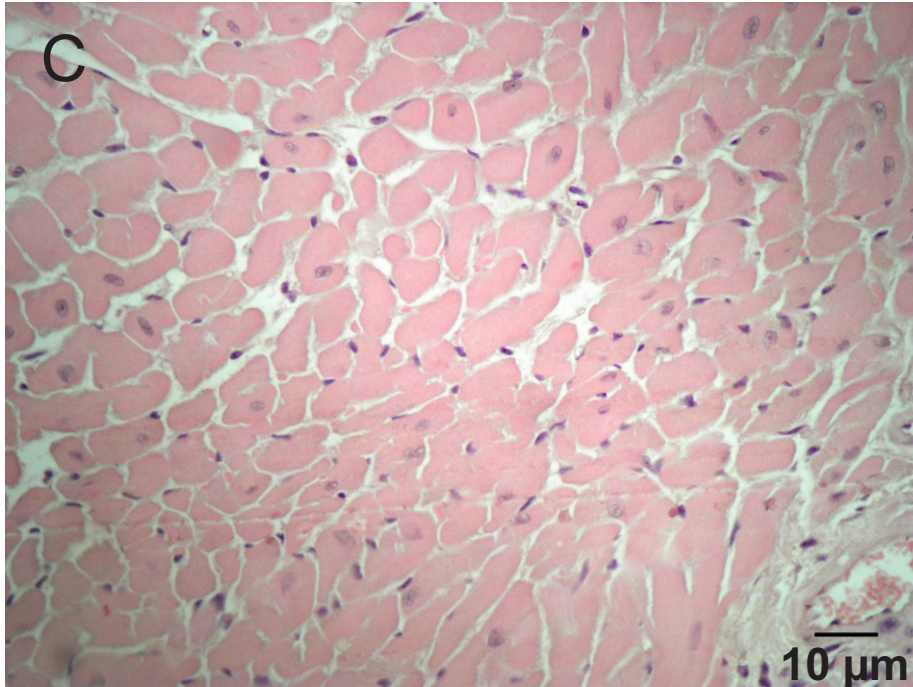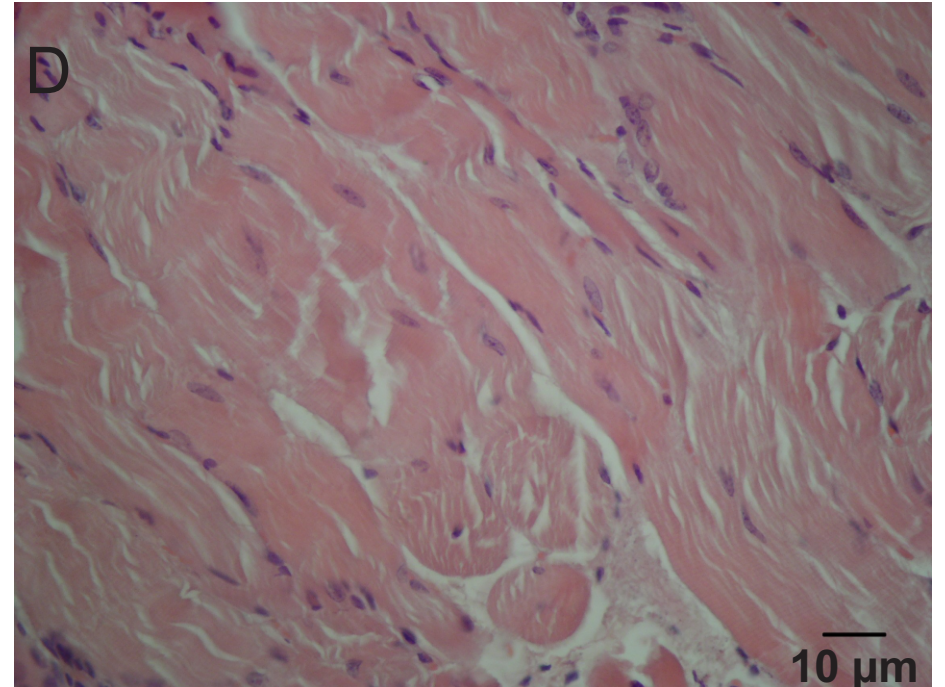

Supplement: Supplementary Figure 1 — Normal tissue analyses at 220 days old animals. (A) Serum enzymatic activity of CK and CK-MB represented as International Units (IU/L); (B) T. cruzi equivalents by qPCR in naïve tissue; Histopathological analysis of non-infected mice, representative image of hematoxylin-eosin stained: (C) Cardiac Tissue; (D) skeletal muscle. Animals showed a conserved architecture of both tissues. Magnification: 40x. [file Image_1.pdf]
